# Effect of titanium implants with strontium incorporation on bone apposition in animal models: A systematic review and meta-analysis

**DOI:** 10.1038/s41598-017-15488-1

**Published:** 2017-11-14

**Authors:** Junyu Shi, Yuan Li, Yingxin Gu, Shichong Qiao, Xiaomeng Zhang, Hongchang Lai

**Affiliations:** Department of Oral and Maxillo-facial Implantology, Shanghai Key Laboratory of Stomatology, Shanghai Ninth People’s Hospital, School of Medicine, Shanghai Jiao Tong University, Shanghai, 200011 China

## Abstract

This systematic review aims to assess the efficacy of titanium (Ti) implant surfaces with or without strontium (Sr) incorporation on osseointegration in animal experimental studies. An electronic search was conducted using databases of PubMed and EMBASE up to November 2016 to identify studies focusing on osseointegration of strontium-modified titanium implants following PRISMA criteria. The primary outcome was the percentage of bone-to-implant contact (BIC) around the implants with or without strontium-modified surface. Of the 1320 studies, 17 studies fulfilling the inclusion criteria were finally included. A random effect meta-analysis was conducted based on BIC in 17 studies, and the results demonstrated considerable heterogeneity (I² = 79%). A sensitivity analysis found that three studies using the same surface modification method were the major source of the heterogeneity. Therefore, exploratory subgroup analysis was performed. Subgroup one including 14 studies showed a standard mean differences (SMD) of 1.42 (95% CI, 1.13–1.71) with no heterogeneity (I² = 0.0%), while subgroup two including the other three studies showed a SMD of 9.49.95% CI, 7.51–11.47) with low heterogeneity (I² = 0.1%). Sr-modified implants in both subgroups showed significantly higher BIC than unmodified implants (P < 0.01). The results showed a statistically significant effect of Sr-modified titanium implant surfaces on osseointegration and bone apposition in animal models.

## Introduction

The long-term success of endosseous implant mainly depends on osseointegration, which is defined as a direct contact between living bone and implant in histological sections. Albrektsson *et al*. defined six factors as pre-requisites for the establishment of osseointegration, implant material, implant design, implant surface, status of bone, surgical technique and the implant loading condition^1^.

Nowadays, titanium and its alloys have been widely applied for fabricating endosseous implant devices such as artificial knees, hip prosthesis and dental implants owing to its excellent biocompatibility, bio-inertness and adequate mechanical properties^2^. The evolution of clinical protocols have not only shortened treatment time but also expanded indications for implant therapy with significant progress of titanium surfaces. Physical, chemical, biological and topographical modifications have been proposed to accelerate bone healing and promote bone formation attempting to reach a rapid, long-living implant anchorage^3–5^. Currently, various studies have demonstrated surface modification with inorganic metal elements such as magnesium (Mg), zinc (Zn), strontium (Sr) incorporation could achieve rapid osseointegration and promote new bone formation^6–8^.

Strontium(Sr) aroused great attention clinically since strontium ranelate (SrRan) had been proved to have significant effect on reducing the risk of fracture in osteoporosis patients^9,10^. Sr, an essential trace element in human body, has been reported to enhance the osseointegration *in vitro* and *in vivo*. *In vitro* studies have found that Sr ion stimulates osteogenic differentiation of mesenchymal stem cells (MSCs) by activating Wnt signaling^11,12^. Sr exerts an inhibitory effect on osteoclast activity and differentiation through the activation of RANK/RANKL pathway and expression of osteoprotegerin (OPG)^13,14^. Moreover, the mechanism is believed to depend on the results of angiogenesis and osteogenesis that Sr facilitates osteogenic differentiation of BMSCs as well as promotes the angiogenic growth factors secretion, which can result in blood vessel formation^15,16^. *In vivo* studies showed that Sr could promote osseointegration both in Sr-loaded implants and via oral administration of SrRan^8,17^. In addition, the beneficial effect on osteogenesis was also observed in Sr-enriched applications such as CaSiO3 ceramics^18^, bioactive glasses^19^ and bone cement^20^.

However, due to the high cost of animal studies, most of the sample sizes in previous studies were limited. Moreover, the methods of adding Sr into implant surfaces varied. Meanwhile, limited high-quality evidence for effect of Sr-modified implant on bone apposition was available. Thus, a systematic review is highly in demand for evaluating the effect of Sr-modified titanium implants surface on enhancing osseointegration.

Therefore, the aim of present review was to systematically analyze the scientific literature reporting the efficacy of treating titanium surfaces with Sr on osseointegration of implants in animal experimental models. A parameter frequently used to quantify osseointegaration is bone-implant contact rate (BIC). It is defined as the ratio between the linear measurement of the surface of implant in direct contact with bone and the total length of the implant profile. As a crucial parameter of histomorphometry, BIC was selected as the primary outcome in the present review. In addition, the null hypothesis was no significant difference of osseointegration could be found between Sr-modified implants and unmodified implants.

## Results

### Study selection

**Figure 1:**
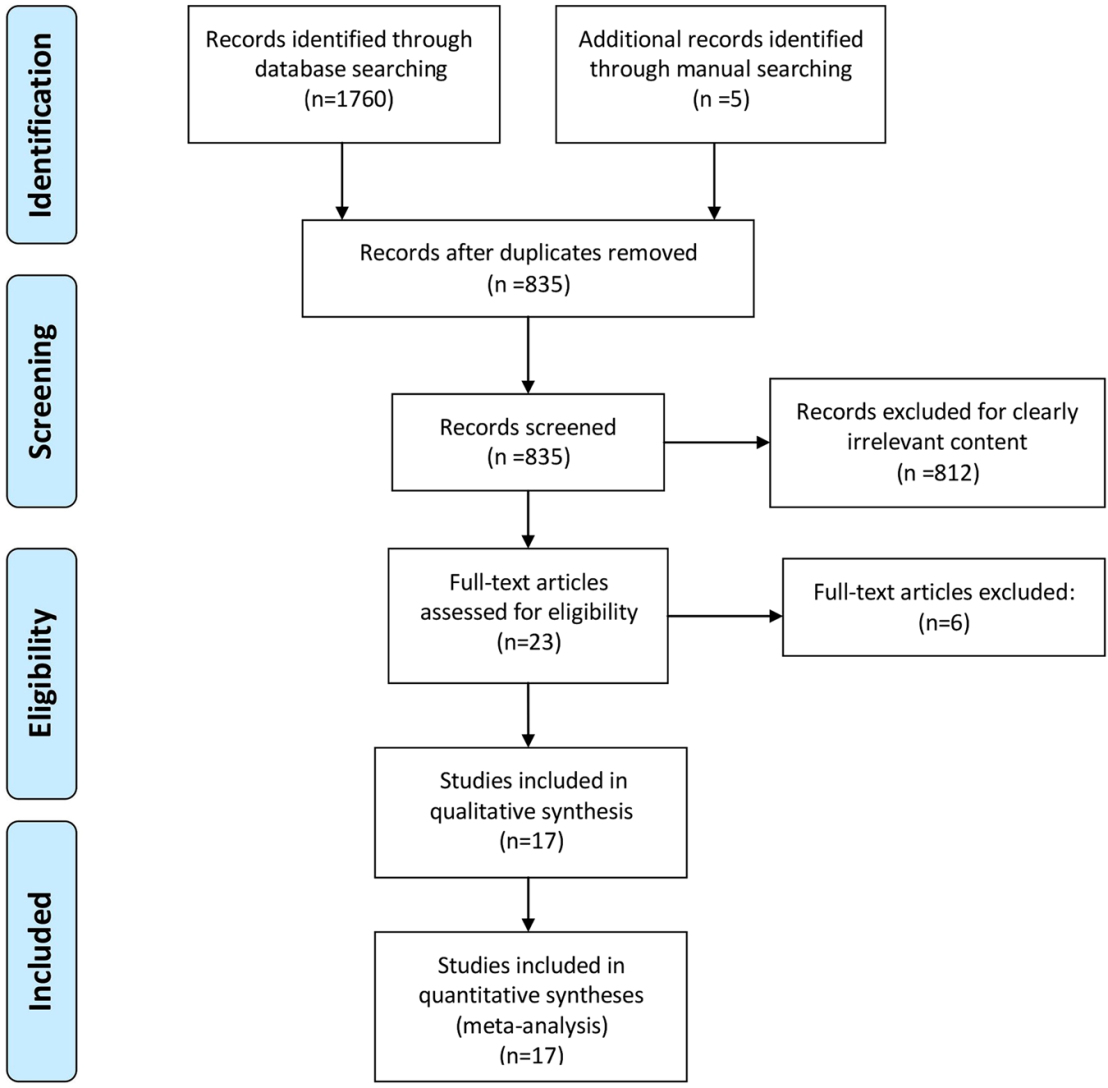
Search flowchart.

Electronic search showed a total number of 1760 titles, of which 835 titles and abstracts were retrieved for possible inclusion after automatic duplication removed. After manual searching bibliographies of the selected studies, 5 studies were added to full-text evaluation. 23 articles were selected for full text evaluation. Three studies were excluded due to no Sr-only experimental group^21–23^. Another three studies were excluded because BIC was not reported^8,24,25^. The final 17 studies^26–42^ were included in this systematic review. The search pathway was showed in Fig. 1. An overview on details about experimental details per study was given in Table 1.

**Table 1:** Characteristics of included studies.

Author	Animal model	Control Groups	Strontium Incorporation Method	Follow-up	Analysis Methods	Outcome
Offermanns *et al*.^26^	30 OVX rats	Smooth-Ti	magnetron sputtering process	6, 12 w	Histomorphometry	BIC BA
Zhang *et al*.^26^	6 beagle dogs	MAO-Ti	micro-arc oxidation	6 w	Histomorphometry	BIC BA
Fan *et al*.^28^	16 male New Zealand white rabbits	SLA Ti	HTP with 0.02 mol/l Sr(OH)_2_·8 H_2_O solution	3, 6 w	RTT Histomorphometry	RTV BIC BA
Dang *et al*.^28^	28 female rats	NT-40 cpTi	HTP with 0.02 M Sr(OH)_2_ 60 mL solution. 200 °C for 1 or 3 h	12 w	pull-out test Micro-CT Histomorphometry	BIC BV/TV, Tb.N Tb.Th, Tb.Sp, Tb.P.F maximal push-out force
Tao *et al*. 2016a	50 female OVX rats	HA-Ti	electrochemical deposition	12 w	pull-out test Micro-CT Histomorphometry	BIC BA BV/TV,Tb.N,Conn.D Tb.Th,Tb.Sp maximal push-out force
Tao *et al*. 2016b	40 female OVX rats	HA-Ti	electrochemical deposition	12 w	pull-out test Micro-CT Histomorphometry	BIC BA BV/TV, Tb.N, Conn.D Tb.Th, Tb.Sp maximal push-out force
Zhang *et al*.^39^	36 female OVX rats	HA-SLA	electrochemical deposition	4, 8, 12 w	Histomorphometry	BIC BA
Li *et al*.^35^	40 female rats	Smooth-Ti	magnetron sputtering	12 w	pull-out test Micro-CT Histomorphometry	BIC BV/TV, Tb.N, Tb.Th, Tb.Sp, maximal push-out force
Offermanns *et al*.^40^	30 female rats	Smooth-Ti	magnetron sputtering process	4 w	Histomorphometry	BIC BA
Andersen *et al*.^33^	20 female rats	Smooth-Ti	magnetron co-sputtering	4 w	Histomorphometry	BIC BA
Zhang *et al*.^36^	12 adult beagle dogs	HT-Ti–6Al–4 V	plasma spray techniques	12 w	pull-out test Micro-CT Histomorphometry	BIC BA BV/TV, Tb.N, Tb.Th maximal push-out force
Park *et al*.^6^	10 male New Zealand white rabbits	SLActive Ti	HTP with mixed solution of SrO and NaOH dissolved in deionized water 180 °C for 2 h	2 w	Histomorphometry RFA	BIC BA ISQ
Yan *et al*.^11^	30 adult rabbits	HA-Ti	micro-arc oxidation	4, 12 w	pull-out test Micro-CT Histomorphometry	BIC BV/TV, Tb.N, Tb.Th, Tb.Sp, maximal push-out force
M. Ballo *et al*. 2012	20 male rats	HA-Ti	biomimetic process	1, 4 w	Histomorphometry	BIC BA
FU *et al*.^42^	10 New Zealand White rabbits	HA-Ti	electrochemical deposition	1, 4, 8 w	Histomorphometry	BIC BA
Li *et al*.^31^	20 female OVX rats	HA-Ti	Sol-gel dip coating with Sr(NO3)2 solution	12 w	pull-out test Micro-CT Histomorphometry	BIC BA BV/TV, Tb.N, Conn.D Tb.Th, Tb.Sp, Tb.P.F maximal push-out force
Park *et al*.^37^	7 New Zealand White rabbits	Ti–6Al–4 V	Hydrothermal treatment	4 w	RTV Histomorphometry	BIC BA RTV
**Implants (n)**	**Implant Dimensions, D × L (mm)**		**Location of Implant Placement**	**Implant Shape**	**Sr-modified Implant Surface Characteristics**	
Ti (60)	1.6 × 5		Tibia	Cylindrical	Ti-Sr-O layer 2000 nm micro-/nano-structures	
Ti alloy (12)	NR		Mandible	NR	Ti-Sr-O layer 1 μm micro-/nano-structures	
cp Ti (64)	4 × 8		Tibia/ Femur	Screw	Ti-Sr-O layer micro-/nano-structures	
cp Ti (112)	3 × 6		Tibia/ Femur	Screw Cylindrical	Sr-loaded nanotubes	
Ti (80)	1.2 × 15		Femur	NR	Sr-HA coatings micro-/nano-size	
Ti (20)	1 × 20		Femur	NR	Sr-HA coatings micro-/nano-size	
SLA Ti (72)	2 × 6		Tibia	NR	Sr-HA coatings micro-/nano-size	
Ti implants (80)	1.5 × 3		Femur	Screw	Ti-Sr-O layer 20–40 nm Sr-loaded nano-textured	
Ti (60)	1.1 × 5		Femur	NR	Ti-Sr-O layer 1200 nm micro-/nano-structures	
Ti (40)	1.1 × 6		Femur	Rod	Ti-Sr-O layer 1000 nm micro-/nano-structures	
Ti–6Al–4 V (48)	3 × 10		Femur	Rod	Sr-HT coatings nano/micron hierarchical structure	
SLA Ti (20)	3.3 × 10		Femur	Screw	Ti-Sr-O layer micro-/nano-structures	
cp Ti (120)	3.75 × 6		Femur	Rod	Sr-HA coatings 32 μm micro-/nano-size	
cp Ti (80)	2 × 2.3		Tibia	Rod	Sr-HA coatings micro-/nano-size	
Ti (20)	4.1 × 8		Femur	Screw	Sr-HA coatings	
Ti (50)	1 × 12		Tibia	Rod	Sr-HA coatings 898 ± 102 nm micro-/nano-size	
Ti–6Al–4 V (64)	2.4 × 8		Tibia/ Femur	Screw	Ti-Sr-O layer 50 nm nanostructure	

OVX: ovariectomized; Micro-CT: microcomputed tomography; BIC: bone to implant contact; BA: bone area; MAO: micro-arc oxidation; RTT: removal torque test; RTV: removal torque value; cpTi: commercially pure titanium; BV/TV: bone volume/total volume; Tb.Sp: trabecular spacing; Tb.N: trabecular number; Tb.Th: trabecular thickness; Conn.D: the mean connective density; Tb.P.F: trabecular pattern factor; NT: nanotube; HTP: hydrothermal process; Sr: strontium; HA: hydroxyapatite; HT: hardystonite; SLA: sandblasted acid-etched

cp Ti: Commercially pure titanium; SLA: sandblasted acid-etched; HA: hydroxyapatites; HT: hardystonite; NR: not reported.

### Risk of bias and quality assessment of included studies

**Figure 2:**
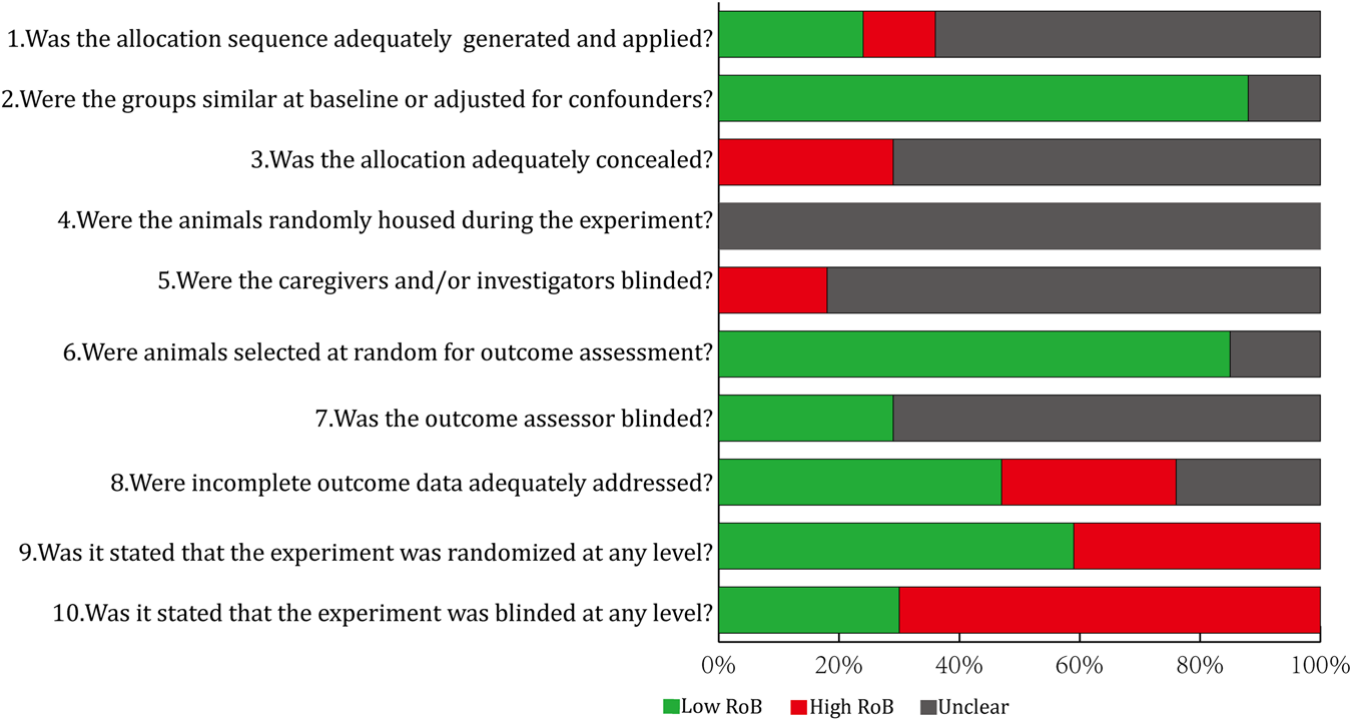
Risk of bias (RoB) measured using the Systematic Review Centre for Laboratory animal Experimentation (SYRCLE) RoB tool, averaged per item.

The results of the risk of bias evaluation of included studies were shown in Fig. 2. For items 9 and 10, 60% of included studies reported the experiment was randomized at some level, while 29% reported blinding at any level during the study.

The ARRIVE criteria of included studies was shown in Table 2. The mean score of all studies was 17.2 (±1.98) out of a maximum of 24. All studies reported adequate information concerning title, abstract, introduction, ethical statement, species, surgical procedure, outcome evaluation, statistical analysis and results. Information regarding experimental animals housing conditions and study limitation were generally inadequate. Animals were randomly allocated to different treatment groups in nine studies (53%). Moreover, five studies reported blinding of assessors to test groups (29%). The 3Rs (in the results section) was not reported in any of studies.

**Table 2:** Checklist of ARRIVE criteria reported by the included studies.

No	ARRIVE criteria	Offer-manns *et al*. (2016)	Zhang *et al*. (2016)	Fan *et al*. (2016)	Dang *et al*. (2016)	Tao *et al*. (2016a)	Tao *et al*. (2016b)	Zhang *et al*. (2015)	Li *et al*. (2015)	Offer-manns *et al*. (2015)	Andersen *et al*. (2013)	Zhang *et al*. (2013)	Park *et al*. (2012)	Yan *et al*. (2012)	M.Ballo *et al*. (2012)	FU *et al*. (2012)	Li *et al*. (2010)	Park *et al*. (2010)
1	Title	1	1	1	1	1	1	1	1	1	1	1	1	1	1	1	1	1
	Abstract																	
2	Species	1	1	1	1	1	1	1	1	1	1	1	1	1	1	1	1	1
3	Key finding	1	1	1	1	1	1	1	1	1	1	1	1	1	1	1	1	1
	Introduction																	
4	Background information	1	1	1	1	1	1	1	1	1	1	1	1	1	1	1	1	1
5	Reasons for animal model	1	0	0	0	1	1	1	0	0	0	0	0	0	0	0	1	0
6	Hypothesis	1	1	1	1	0	1	0	1	1	0	1	0	1	0	1	0	1
	Methods																	
7	Ethical statement	1	1	1	1	1	1	1	1	1	1	1	1	1	1	1	1	1
8	Randomization of animal	0	0	0	1	0	0	1	1	1	1	1	0	1	1	0	1	0
9	Blinding of assessor	0	1	0	0	1	1	0	0	0	0	1	0	0	0	0	1	0
10	Anaesthesia	1	1	1	1	1	1	1	1	1	1	1	1	1	1	1	1	1
11	Antibiotics	1	0	1	0	0	0	1	1	1	1	0	0	1	1	0	1	1
12	Analgesia	0	0	0	0	0	0	0	0	1	1	0	0	0	0	0	1	0
13	Surgical procedure	1	1	1	1	1	1	1	1	1	1	1	1	1	1	1	1	1
14	Reporting species	1	1	1	1	1	1	1	1	1	1	1	1	1	1	1	1	1
15	Housing conditions	0	0	0	1	1	1	0	0	0	0	0	0	0	0	0	1	0
16	Implant randomization	1	1	1	0	1	1	0	1	0	1	1	0	0	1	0	0	1
17	Statistical methods	1	1	1	1	1	1	1	1	1	1	1	1	1	1	1	1	1
	Results																	
18	Results reported	1	1	1	1	1	1	1	1	1	1	1	1	1	1	1	1	1
19	Standard error/confidence interval	1	1	1	1	1	1	1	1	1	1	1	1	1	1	1	1	1
	Discussion																	
20	Interpretation	1	1	1	1	1	1	1	1	1	1	1	1	1	1	1	1	1
21	Study limitations	0	0	1	0	1	1	1	1	0	0	0	0	1	1	0	1	1
22	3 Rs reported	0	0	0	0	0	0	0	0	0	0	0	0	0	0	0	0	0
23	Relevance to humans	1	0	1	1	0	0	1	0	1	0	1	0	1	0	0	1	0
24	Funding	1	1	1	1	1	1	1	1	1	1	1	1	1	0	0	1	1
Total score		18	16	18	18	18	19	18	18	18	15	18	13	18	16	13	21	17

### Bone to implant contact

**Figure 3:**
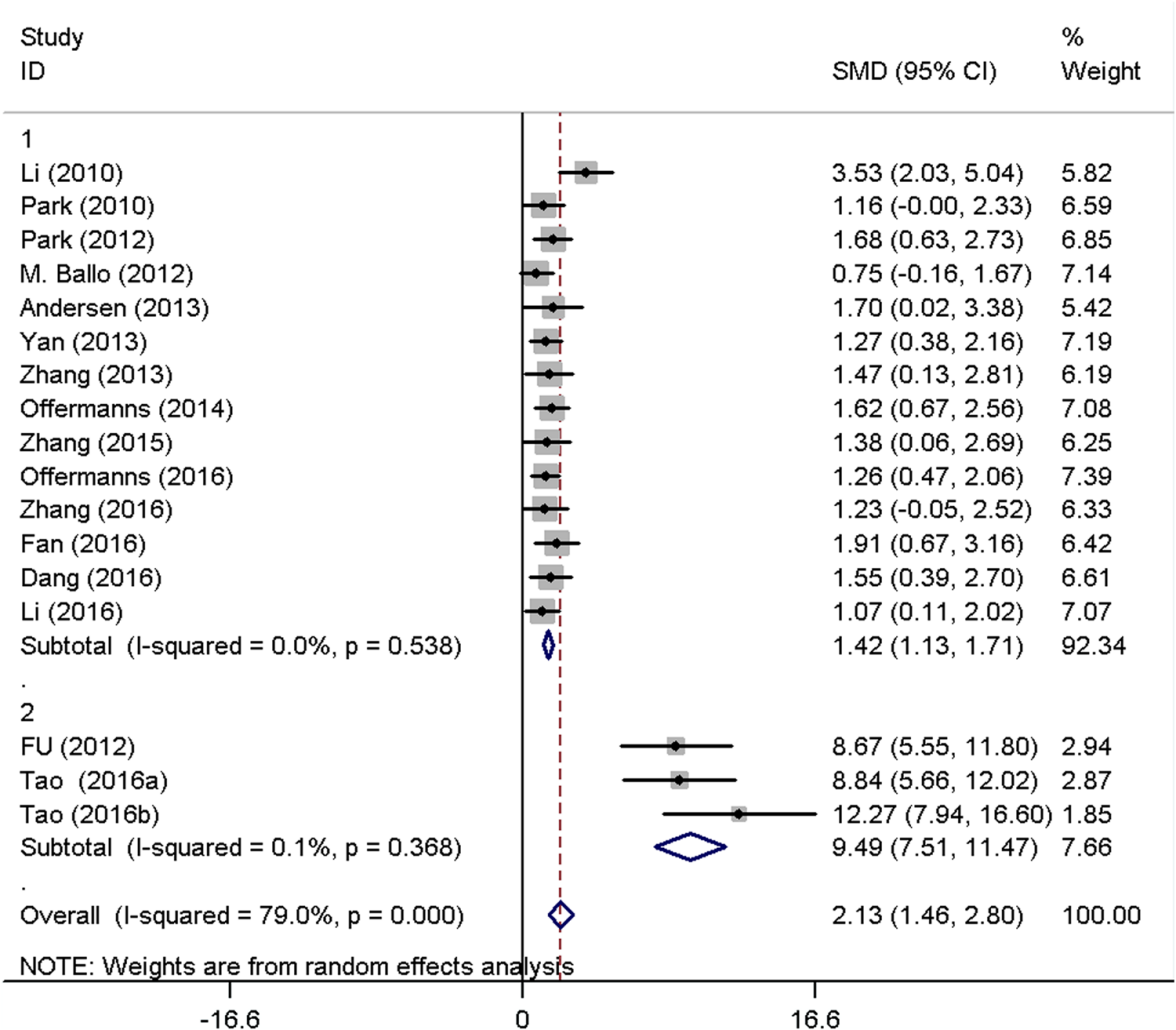
Forest plots of bone to implant contact (BIC).

**Figure 4:**
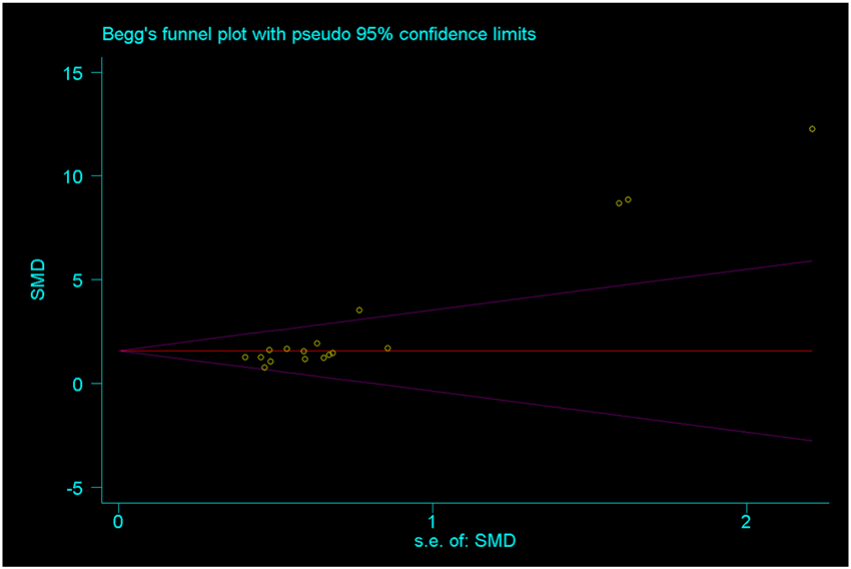
Begg’s funnel plot of included studies.

All included studies measured the effect on BIC around implants with or without Sr-enriched surface. A random effect meta-analysis was conducted based on BIC in 17 studies, and the overall results demonstrated considerable heterogeneity (I² = 79%). A sensitivity analysis found that three studies using the same surface modification method were the major source of the heterogeneity. Therefore, exploratory subgroup analysis was performed. The subgroup1 including 14 studies showed a standard mean differences (SMD) of 1.42 (95% CI, 1.13–1.71) with no heterogeneity (I² = 0.0%), while subgroup2 including the other three studies showed a SMD of 9.49 (95% CI, 7.51–11.47) with low heterogeneity (I² = 0.1%) (Fig. 3). The Sr-modified implants in both subgroups showed significantly higher BIC than unmodified implants (P < 0.01). However, high publication bias was found in the present study. (Begg, p = 0.039; Egger, p = 0.000) (Fig. 4.).

### Bone area ratio

14 of 17 studies^26–28,30–34,37–42^ calculated the percentage of new bone area (BA) of peri-implants. No meta-analysis could be conducted due to the considerable heterogeneity. 10 studies reported that significant higher bone area could be observed around strontium modified implants than those implants without strontium incorporation. However, no significant difference in bone area was reported in other four studies^28,30,37,42^.

### Micro-CT evaluation and biomechanical test

Micro-computed tomography (micro-CT) evaluation was performed in seven of included studies^29,31,32,34–36^. In quantitative assessment, four studies^31,32,34,38^ showed Sr-enhanced implants demonstrated stronger effect on all micro-CT parameters including bone volume per total volume (BV/TV), 3D bone distribution such as trabecular number (Tb.N), thickness (Tb.Th), and/or spacing (Tb.Sp) and/or the connective density (Conn.D) than control implants, while other three studies found no statistically significant changes in some parameters.

Nine of included studies^28,29,31,32,34–38^ reported the results of biomechanical test including pull-out test or removal torque testing. All studies revealed a significant increase in implant fixation, with removal torque value, the maximal push-out force and/or the ultimate shear strength markedly raised compared to control groups.

## Discussion

The primary finding of this meta-analysis was that Sr-modified implant surfaces significantly increased the percentage of BIC. Therefore, the null hypothesis should be rejected.

To our knowledge, this is the first systematic review to assess the effect of Sr-modified implants on enhancing osseointegration and bone apposition in animal experimental studies. To quantify the potential effect of Sr-containing surfaces on peri-implant bone apposition, a meta-analysis of BIC was performed. Overall, the results of subgroup meta-analysis revealed that titanium implants with strontium incorporation demonstrated significantly better BIC than unmodified implants in small (rats, rabbits) and large (dogs) animals. The result verified the expectation *in vitro* studies that the Sr-containing titanium surface is expected to shorten bone healing period and enhance implant osseointegration^43,44^. Similarly, this result was in agreement with studies for appraising the efficiency of Sr-modified magnesium (Mg) based implant^45,46^. In these studies, Sr-enriched implant showed significantly higher percentage of BIC than that of pure Mg or Mg alloy based implant.

It is worth mentioning that subgroup two reported Sr-modified surfaces had significant effect on BIC and the difference between implants with and without strontium incorporation was more significant than that in subgroup one. The identical electrochemical deposition process was applied to incorporate strontium-substituted hydroxyapatite (Sr-HA) into surface in these three studies indicating the heterogeneity may be caused by method of surface modification. It has been reported that an electrochemical process can produce a homogeneous 2- to approximately 3 μm HA coating and nano-hydroxyapatite (nano-HA) on the metallic substrate surface^47^. Yang *et al*.^48^ reported that significant superiority of osseointegration and bone apposition was found when electrochemical deposition method was used. However, it is difficult to determine the best surface modification methods due to the limited information.

New bone area plays an important role in evaluating the osteoconductive property of biomaterials. The new mineralized bone tissue area inside all the implant threads was measured to evaluate the percentage of bone area. Several studies have demonstrated that Sr-containing biomaterials could increase new bone apposition. Studies for evaluating Sr-incorporated bioactive glass scaffolds and bone cement in impaired bone found that materials were covered by more new bone than unmodified groups^49,50^. In this review, ten studies using rat animal model reported significantly higher BA in Sr-modified implants than unmodified implants, while the other four studies used rabbit animal model reporting no significant difference in BA. The difference may be attributed to the different animal model, which implies different dynamics of bone formation especially in early healing intervals^51^. Therefore, additional preclinical and clinical studies should be performed to assess the effect of Sr-modified implant on bone apposition and osseointegration.

The 3-D micro-CT image clearly provides the information of bone–implant interface and trabecular microstructure of peri-implant bone tissue from both qualitative and quantitative perspectives. Seven included studies^29,31,32,34–36,38^ performed CT evaluation and four of them showed significantly improved BV/TV, Tb.N, Tb.Th, Tb.Sp and/or Conn.D. Other three studies found the parameters of CT evaluation were partially improved by Sr-modified titanium surface. Possible reasons that cause the disparity could be the different surface topography and Sr concentration of titanium implant surfaces. Furthermore, biomechanical testing further demonstrated a significant increase in implant fixation. It was apparent to be verified by the improved trabecular bone microarchitecture together with increased BIC and BA around the implant.

All the included studies reported that the improved implant osseointegration and bone apposition was attributed to the released Sr ions and modified surface topography. Actually, the exact mechanism regarding bone remodeling effect of Sr has not been clearly understood. Recently, it has been suggested that the possible mechanism of Sr relies on the calcium-sensing receptor (CaR) which is expressed in several types of pre-osteoblastic cells and bone marrow stromal cells^52^. Through a CaR-mediated mechanism, Sr is reported to increase bone apposition by promoting pre-osteoblastic cell proliferation and differentiation, reducing osteoclast differentiation, enhancing matrix mineralization^52,53^. In addition, the surface topography changed by strontium incorporation process also contributed to bone-implant integration. Three of included studies revealed that micro/nanoscale topography enhanced new bone apposition and osseointegration of Sr-modified implants or had a synergistic effect with released Sr ions^28,29,35^. Hence, in-depth investigations are required to isolate the pure and independent effect of strontium or surface topography on improving bone-implant integration.

The present systematic review had several limitations. Firstly, the follow-up of included studies ranged from 1 week to 12 weeks, so it remained unclear whether the osseointegration of Sr-incorporated Ti implants would be a stable anchorage, which could contribute to their long-term survival. Secondly, high publication bias (Begg, p = 0.039; Egger, p = 0.000) was found in Begg’s and Egger’s test. Therefore, the results need to be interpreted with caution. Thirdly, none of the implants included in the present study were loaded. Thus, future studies should evaluate the effect of Sr-incorporated Ti implants under loading conditions.

## Conclusion

Based on available evidence so far, it can be concluded that Sr-modified titanium implants could enhance osseointegration and new bone formation of peri-implant area in animal models. Nonetheless, future clinical investigations are needed to verify the safety and effectiveness of Sr-modified implants.

## Methods

### PICO

According to the Preferred Reporting Items for Systematic Reviews and Meta-Analyses (PRISMA) guidelines^54^, a specific question was identified based on the Participants, Interventions, Control, Outcomes (PICO) principle. The focused question was, “Does incorporating strontium into titanium implant surfaces influence the osseointegration?”

(P) Participants: Subjects received endosseous implantation.

(I) Interventions: Implants with strontium incorporation.

(C) Control: Implants without strontium incorporation.

(O) Outcome: BIC, BA and results from biomechanical test and micro-CT evaluation.

### Search strategy and study selection

Databases of PubMed and EMBASE were searched up to November 2016 for relevant articles published, using search terms “strontium”, in combination with “osseointegration”, “bone apposition”, “osteogenic”, “osteogenesis”, “new bone formation”, “bone to implant contact” and “bone regeneration”. Additionally, bibliographies of the selected studies and relevant review articles were also scrutinized for cross-references. Titles and abstracts of searches were initially screened by two authors (SJY, LY). Uncertainty in the determination of eligibility was resolved by discussion. Two authors reviewed full-text articles independently and final inclusion was based on the inclusion criteria.

### Inclusion and exclusion criteria

The inclusion criteria for the study selection were:

1. Studies regarding titanium implants modified with strontium;
2. Studies reporting the percentage of bone-to-implant contact of Sr-modified and unmodified implants;
3. Studies with a minimum of 3 implants/group;

The exclusion criteria for the study selection were:

1. *In vitro* studies;
2. Studies assessing the combined effect of Sr and other inorganic elements (e.g. Ag, phosphate) modified surface without strontium-only test group.

### Risk of bias and quality assessment

The risk of bias (RoB) of included studies was assessed using the SYRCLE RoB tool for animal studies^55^. The tool, which aims to assess methodological quality, was adapted to appraise bias in animal studies. RoB was evaluated by providing a response of “high”, “low” or “unclear” in each of the 10 items. As reported in a previous review, a modified RoB tool was used in which items 9 and 10 were adjusted to include information on whether the experiment was randomized or blinded at any level (Fig. 2.).

Reporting quality of the included studies was assessed based on a modified ARRIVE guidelines in which a checklist of 24 items was included^56^. Each item was judged as “0” (not reported) or “1” (reported). The total score of each of included studies was also recorded (Table 2).

### Data extraction

Two independent reviewers (SJY, LY) extracted data from the full-texts of selected articles. General information, animal parameters (total number, species), methods of strontium incorporation, evaluation time points, analysis methods and outcomes and implant parameters (total number, material, length, diameter, shape, location and surface characteristics of test and control implants) were retrieved. The primary outcome and secondary outcomes were extracted (Tables 3 and 4). If data were only expressed graphically, numerical values were requested from the authors, and if a response was not received, digital ruler software was used to measure graphical data (ImageJ, National Institutes of Health, Bethesda, MD).

**Table 3:** Micro-CT values of included studies.

Parameters	Dang *et al*. (2016)		Tao *et al*. (2016)		Tao *et al*. (2016)		Li *et al*. (2015)		Zhang *et al*. (2013)		Yan *et al*. (2013)		Li *et al*. (2010)	
	C	T	C	T	C	T	C	T	C	T	C	T	C	T
BV/TV (%)	16.7 ± 0.8	15.9 ± 0.2	23.8 ± 1.2	40.2 ± 2.4*	23.8 ± 1.2	28.6 ± 1.2*	36.4 ± 3.6	39.8 ± 5.3*	27.0 ± 6.2	43.2 ± 6.4**	38.6 ± 2.9	42.4 ± 3.3**	24.7 ± 4.9	42.9 ± 6.7*
Tb.N(mm-1)	4.1 ± 0.1	4.3 ± 0.0	226.2 ± 7.3	503.9 ± 15.4*	226.2 ± 7.3	382.2 ± 13.8*	3.3 ± 0.2	3.8 ± 0.2	2.1 ± 0.4	3.7 ± 0.6**	2.8 ± 0.3	3.2. ± 0.2	2.3 ± 0.4	4.9 ± 0.7**
Tb.Th (μm)	0.044 ± 0.0	0.046 ± 0.0	77.6 ± 3.7	116.3 ± 4.1*	77.6 ± 3.7	90.4 ± 3.5*	108.2 ± 19.4	132.5 ± 21.1	163.1 ± 26.9	225.9 ± 34.3**	140.0 ± 10.4	148.3 ± 22.7	80.2 ± 9.7	102.3 ± 12.6*
Tb.Sp (μm)	0.21 ± 0.01	0.19 ± 0.0	428.98 ± 25.1	291.59 ± 10.9*	428.98 ± 25.1	364.84 ± 13.8*	276.6 ± 52.8	200.1 ± 51.2	NR		220.1 ± 32.4	183.9 ± 27.3**	425.8 ± 50.2	342.1 ± 42.4*
Conn.D(mm-3)	NR		25.19 ± 1.1	42.97 ± 2.4*	25.19 ± 1.1	29.03 ± 1.1*	NR		NR		NR		25.9 ± 3.8	40.3 ± 6.7*
Tb.P.F(mm-1)	13.5 ± 0.3	11.2 ± 0.5**	NR		NR		NR		NR		NR		NR	
Number of implants/group	4		9		10		10		6		12		10	

C: control group; T: test group; BV/TV: bone volume/total volume; Tb.Sp: trabecular spacing; Tb.N: trabecular number; Tb.Th: trabecular thickness; Conn.D: the mean connective density; Tb.P.F: trabecular pattern factor; NR: not reported; Data were expressed as mean ± SD; *p Value < 0.05; **p Value < 0.01.

**Table 4:** Bone-to-Implant Contact (BIC) Values.

Author	Year	Number of implants per group	BIC		BA		Biomechanical test	
			control	test	control	test	control	test
Offermanns *et al*.^#^	2016	15	65.2 ± 10.4	78.2 ± 9.6^*^	23.8 ± 4.2	44.6 ± 9.4^*^	NR	
Zhang *et al*.	2016	6	49.6 ± 6.5	58.7 ± 7.1^**^	29.5 ± 8.6	60.8 ± 10.3^**^	NR	
Fan *et al*.	2016	8	64 ± 5.9	77.1 ± 7.0^*^	6.3 ± 6.5	0.8 ± 1.7	★41.1 ± 8.2	★56.8 ± 18.6^*^
Dang *et al*.	2016	8	59.5 ± 3.2	63.2 ± 0.1^*^	NR		10.8 ± 1.2	24.6 ± 2.4^**^
Tao *et al*.	2016a	10	17.2 ± 2.0	37.1 ± 2.3^*^	18.3 ± 0.9	33.7 ± 2.1^*^	121.2 ± 11.4	168.9 ± 22.2^*^
Tao *et al*.	2016b	10	34.6 ± 0.7	62.5 ± 3.0^*^	31.3 ± 0.8	47.32 ± 2.3^*^	126.3 ± 12.3	219.5 ± 21.8^*^
Zhang *et al*.	2015	6	51 ± 6.6	58.6 ± 2.9^*^	28.6 ± 6.2	44.5 ± 9.7^*^	NR	
Li *et al*.	2015	10	39.7 ± 6.0	46.1 ± 5.5^*^	NR		108.9 ± 46.5	136.9 ± 21.7^*^
Offermanns *et al*.^#^	2015	12	27.8 ± 1.7	45.6 ± 2.2^**^	17.8 ± 2.2	24.4 ± 7.8^*^	NR	
Andersen *et al*.^#^	2013	5	0 ± 1.2	18 ± 14.3^**^	0 ± 5.1	22 ± 7.3^*^	NR	
Zhang *et al*.	2013	6	37 ± 8.7	51.2 ± 9.1^*^	40.6 ± 5.7	55.2 ± 9.8^*^	229.08 ± 59.0	388.84 ± 100.5^**^
Park *et al*.	2012	10	75.4 ± 5.4	84.6 ± 5.1^**^	57.3 ± 9.4	60.9 ± 10	NR	
Yan *et al*.^#^	2012	12	58.8 ± 4.7	65.1 ± 4.9^*^	NR		119.23 ± 3.9	142.31 ± 9.6^*^
M. Ballo *et al*.	2012	10	26.4 ± 10.7	35.7 ± 12.9^*^	25.7 ± 2.9	32.9 ± 9.3^*^	NR	
FU *et al*.	2012	10	72.2 ± 12.8	87.7 ± 2.8^*^	9.3 ± 0.5	9.8 ± 1.5	NR	
Park *et al*.	2010	7	47.3 ± 10.4	60.1 ± 10.2^*^	46.7 ± 10.7	46.6 ± 6.5	★1.6 ± 0.9	★2.6 ± 1.2^*^
Li *et al*.	2010	10	42.5 ± 4.1	63 ± 6.7^*^	25.2 ± 2.2	42.73 ± 4.3^*^	38.7 ± 5.3	80.2 ± 9.9^**^

Values are shown as mean ± SD; ^#^values are presented as median value ± interquartile range;

BIC, BA values in %; pull-out test values in N; ★removal torque testing values in Ncm;

*p Value < 0.05; **p Value < 0.01; NR: not reported.

### Statistical analysis

The primary and secondary outcomes were present in descriptive statistics. The standardized mean differences, together with 95% confidence intervals, were analyzed using random-effect model. Heterogeneity was tested using the *I* ^2^ statistic to describe the proportion of total variation. Values of 25, 50 and 75% were regarded as low, moderate and considerable heterogeneity, respectively^57^. When the value was >50%, qualitative analysis was conducted. A forest plot was generated, and heterogeneity was calculated by use of the statistical software package STATA (v11.0; StataCorp, College Station, TX). A p value < 0.05 was considered to indicate statistical significance, unless specified otherwise.

Potential publication bias was assessed using Begg’s funnel plots and Egger’s test at the p < 0.10 level of significance.
